# Construction and Validation of an Occupational Risks Scale for Intra-hospital Nursing Staff* ** ***

**DOI:** 10.17533/udea.iee.v41n2e16

**Published:** 2023-08-29

**Authors:** Katya Cuadros-Carlesi, Carlos Henríquez-Roldán, Elena Meneses-Ciuffardi, Jaime Fuentes-Ibáñez, Paola Ruiz-Araya

**Affiliations:** 1 PhD in Nursing, Nursing career academician, Universidad Viña del Mar, Chile. Email: katya.cuadros@uvm.cl. https://orcid.org/0000-0003-4751-815X Universidad de Viña del Mar Universidad Viña del Mar Chile katya.cuadros@uvm.cl; 2 PhD in Biostatistics, Academician in the Faculty of Sciences, Universidad de Valparaíso, Chile. Email: carlos.henriquez@uv.cl https://orcid.org/0000-0001-6616-1243 Universidad de Valparaíso Faculty of Sciences Universidad de Valparaíso Chile carlos.henriquez@uv.cl; 3 Master’s in Organizational Development and Human Resources, Academician in the Faculty of Psychology, Universidad UNIACC, Santiago, Chile. Email: elena.meneses@gmail.com https://orcid.org/0000-0001-6616-1243 Faculty of Psychology Universidad UNIACC Santiago Chile elena.meneses@gmail.com; 4 Master’s in Management Consulting and Development, Independent researcher, Hospital de Curacaví, Curacaví, Chile. E-mail: jaimefuentesi@gmail.com http://orcid.org/0000-0002-3536-3798 Hospital de Curacaví Curacaví Chile jaimefuentesi@gmail.com; 5 PhD in Projects. Nursing career academician, Universidad Viña del Mar, Chile. E-mail: paola.ruiz@uvm.cl http://orcid.org/0000-0001-9485-598X Universidad de Viña del Mar Nursing career academician Universidad Viña del Mar Chile paola.ruiz@uvm.cl http://orcid.org/0000-0001-9485-598X

**Keywords:** occupational risks, occupational health nursing, nursing staff, hospitals, surveys and questionnaires, riesgos laborales, enfermería del trabajo, personal de enfermería, hospitales, encuestas y cuestionarios, riscos ocupacionais, enfermagem do trabalho, recursos humanos de enfermagem, hospitais, inquéritos e questionários

## Abstract

**Objective:**

To construct and evaluate initial validity indicators of an instrument on occupational risks for hospital nursing staff.

**Methods:**

A methodological study was conducted in four Chilean hospitals. The study was carried out in three stages: (i) integrative literature review on risk assessment instruments for nursing; (ii) descriptive qualitative study on 113 health professionals to identify their work conditions and experiences regarding occupational risks and construct three instruments proposals for nursing managers, clinical nurses, and technicians; and (iii) validity and reliability study of the three instruments in 503 nurses and nursing technicians. To collect the data from the qualitative study, individual interviews, focal groups, and non-participant observation were conducted. The data were analyzed thematically into predefined risk categories. Content validation was performed through expert judgment, and exploratory factor analysis of principal components was conducted for the preliminary construct validity study. Cronbach's alpha was used as an indicator of internal consistency.

**Results:**

A total of 128 items were identified, distributed across 11 categories and 25 subcategories of occupational risks for the three instruments derived from the original proposal. After expert validation, pilot study, and instrument administration, Cronbach's alpha values between 0.88 and 0.93 were obtained. Exploratory factor analysis distinguished eight to eleven components, with unsatisfactory goodness-of-fit indicators.

**Conclusion:**

The instruments demonstrated good parameters of content validity and reliability, although their construct validity needs further improvement.

## Resumen

**Objetivo:**

Construir y evaluar indicadores de validez inicial de un instrumento sobre riesgos laborales del personal de enfermería intrahospitalario.

**Métodos:**

Estudio metodológico, participaron cuatro hospitales chilenos. Se desarrolló en tres etapas: (i) revisión integradora de la literatura sobre instrumentos de evaluación de riesgos en enfermería; (ii) estudio cualitativo descriptivo en 113 profesionales de salud para identificar sus condiciones laborales y vivencias respecto a los riesgos laborales y construir tres propuestas de instrumentos para jefaturas de enfermería, enfermeros clínicos y técnicos; y (iii) estudio de validez y confiabilidad de los tres instrumentos en 503 enfermeros y técnicos en enfermería. Para la recolección de datos del estudio cualitativo se realizaron entrevistas individuales, grupos focales y observación no participante. Los datos fueron analizados temáticamente en categorías de riesgos predefinidas. La validación de contenido se realizó a través del juicio de expertos y para el estudio preliminar de validez de constructo se hizo análisis factorial exploratorio de componentes principales. Como indicador de la consistencia interna se aplicó Alfa de Cronbach.

**Resultados:**

Se identificaron 128 ítems para 11 categorías y 25 subcategorías de riesgos laborales para tres instrumentos derivados del originalmente propuesto. Luego de la validación por expertos, el estudio piloto y la aplicación de los instrumentos, se obtuvieron valores de alfa de Cronbach entre 0.88 y 0.93. El análisis factorial exploratorio distinguió ocho a once componentes, no lográndose buenos indicadores de bondad de ajuste.

**Conclusión:**

Los instrumentos presentan buenos parámetros de validez de contenido y confiabilidad, debiéndose perfeccionar su validez de constructo.

## Resumo

**Objetivo:**

Construir e avaliar indicadores de validade inicial de um instrumento sobre riscos ocupacionais da equipe de enfermagem intra-hospitalar.

**Métodos:**

Estudo metodológico, quatro hospitais chilenos participaram. Foi desenvolvido em três etapas: (i) revisão integrativa da literatura sobre instrumentos de avaliação de risco em enfermagem; (ii) estudo descritivo qualitativo em 113 profissionais de saúde para identificar suas condições de trabalho e experiências em relação aos riscos ocupacionais e construir três propostas de instrumentos para chefes, enfermeiros assistenciais e técnicos de enfermagem; e (iii) estudo de validade e confiabilidade dos três instrumentos em 503 enfermeiros e técnicos de enfermagem. Para a coleta de dados do estudo qualitativo, foram realizadas entrevistas individuais, grupos focais e observação não participante. Os dados foram analisados tematicamente em categorias de risco pré-definidas. A validação de conteúdo foi realizada por meio de julgamento de especialistas e para o estudo preliminar de validade de construto foi realizada uma análise fatorial exploratória de componentes principais. Como indicador de consistência interna, foi aplicado o Alfa de Cronbach.

**Resultados:**

Foram identificados 128 itens para 11 categorias e 25 subcategorias de riscos ocupacionais para três instrumentos derivados do originalmente proposto. Após a validação por especialistas, o estudo piloto e a aplicação dos instrumentos, foram obtidos valores de alfa de Cronbach entre 0.88 e 0.93. A análise fatorial exploratória distinguiu de oito a onze componentes, não alcançando bons indicadores de qualidade de ajuste.

**Conclusão:**

Os instrumentos apresentam bons parâmetros de validade de conteúdo e confiabilidade, e sua validade de construto deve ser aprimorada.

## Introduction

Intrahospital nursing work is an activity that entails an overload inherent to the nature of its activities and the environment, taking place under complex working conditions.^1^^-^^3^ Continuous contact with patients and their families, performing procedures, nightwork, confrontation with suffering, pain, and death within a stressful context of staff shortage, and a continuous process of decision-making under pressure^4^^,^^5^ exposes the nursing staff to multiple risks that can trigger the occurrence of accidents and occupational diseases.^6^^-^^8^ Work shift, especially night shifts, derives into chronodisruptive disruptions, eating disorders, insomnia, and anxiety.^9^ Besides, this work system limits the time dedicated to their families, provoking conflicts between work and personal life.^10^ Additionally, this work system limits the time dedicated to their families, causing conflicts between work and personal life. Furthermore, the handling of chemical substances leads to a wide range of health problems. ^11^^-^^14^. In addition, the need to remain standing for long periods of time or the mobilization of patients and heavy objects may cause musculoskeletal disorders.^15^

Stress and burnout have also been identified as relevant risks associated with patient care and the organizational factors that impact the performance of nursing duties.^16^ These factors include the performance of additional tasks outside the nursing scope and the staff shortage.^17^ The COVID-19 pandemic added another variable, over-demanding nursing staff in an environment of uncertainty provoking negative effects on their mental health.^18^^,^^19^ Finally, violence in the workplace, principally from patients or their relatives, emerges as a challenging issue to be prevented and managed, putting the physical and emotional integrity of healthcare workers at risk.^20^

Given that the health protection and well-being of nursing workers has implications for the achievement of positive outcomes for the staff, patients, organizations, and health systems,^21^ generation of strategies aimed to improve systematically the safety of working conditions is especially relevant to guarantee a safe working environment. In this sense, it turns out crucial to identify and evaluate risks to generate interventions that improve well-being and work safety. Although there are tools available to asses nursing occupational risks, these have been developed in other countries^22^ and have not been validated in Chile. Furthermore, they measure specific risks, without considering a comprehensive perspective or specific aspects of different nursing and nursing technicians (NTEs). Regarding the lack of knowledge about occupational risks according to the area of nursing practice, the Chilean Social Security Superintendent initiated a research call to develop an instrument to measure occupational risks in intrahospital nursing teams. Therefore, this study aims to construct and validate an assessment instrument for occupational risks among nursing personnel working in Chilean hospitals.

## Methods

A methodological study with a mixed, qualitative, and quantitative design was conducted in four hospitals located in the provinces of Aconcagua and Los Andes in the region of Valparaíso, Chile. The participating hospitals were San Juan de Dios Hospital in Los Andes (172 beds), San Camilo Hospital ( 232 beds), Philippe Pinel Hospital (354 beds), and San Francisco de Llay-Llay Hospital (45 beds). The first two are of high complexity, while the third corresponds to a medium-complexity psychiatric hospital. Finally, the Llay-Llay Hospital is a low-complexity hospital. The research was conducted in three phases; the first involved an integrative literature review with the aim of knowing and guiding the construction of risk categories as a basis for developing the assessment tool. The second consisted of a descriptive qualitative study to know the working conditions of the nursing staff and their exposure to risks in hospitals. Finally, the third stage involved a quantitative study that aimed to validate the instrument and establish its psychometric properties.

### Stage 1. Integrative literature review

The integrative literature review regarding occupational risks and risk assessment tools for nursing staff was carried out between September and November 2020. PubMed, Ebsco Host, Scopus, Cochrane Library, and Scielo databases were reviewed to answer the following guiding question: "What evidence is available in the literature regarding the occupational risks of nursing personnel working in hospitals and the tools to assess these risks?" Spanish and English DeCS/MeSH keywords were used, including nursing, occupational health, hospitals, occupational risks, risk assessment, occupational accidents, psychological stress, emotional exhaustion, workplace violence, circadian rhythm sleep disorders, shift work schedule, leadership, emotional intelligence, and pandemic. Primary studies, systematic reviews, integrative reviews, and meta-analyses from the last 15 years were searched, combining different keywords to ensure comprehensive results. This preliminary stage not only provided a deep understanding of a wide range of publications related to the research topic but also guided the construction of categories and subcategories of occupational risks based on a preliminary classification according to their nature for use in the next stage.

### Stage 2. Qualitative study

This stage was carried out between January and September 2021. Due to the COVID-19 pandemic, the work modality was adjusted, conducting part of the activities remotely using the Zoom^®^ platform. The participants included nursing managers, supervisory nurses, clinical nurses, administrative nurses, nursing technicians (NTEs), occupational risk officers, and occupational psychologists. The inclusion criteria for the nursing staff established having work experience of at least two years in the clinical unit. While for the risk prevention managers and occupational psychologist, the inclusion criteria established having work experience of at least one year in the hospital. The sample size for each hospital was determined by convenience in collaboration with the nursing management of each hospital, based on the inclusion criteria and availability of participants at the data collection time. The sample consisted of 113 participants, including four hospital nursing managers, 12 supervisory nurses, 47 clinical nurses, 45 NTEs, four occupational risk officers, and one occupational psychologist.

Data collection involved focus groups, individual interviews, and non-participant observation of the daily activities of nurses and NTEs. Guidelines were developed for conducting interviews and focus groups. Ten face-to-face interviews, 20 video conference interviews, and 11 hybrid format focus groups were conducted, including five with clinical nurses and six with NTEs. Both interviews and focus groups were guided by two occupational psychologists with experience in qualitative research. Prior to the activities, informed consent was obtained from the participants.

The interviews were conducted with four nursing managers, 12 supervisory nurses, eight clinical nurses, one nursing technician (who couldn't participate in the focus group), four occupational risk officers, and one occupational psychologist. The interviews took place in closed offices within their respective units. Before each interview, feasibility was checked, and the objective of the activity was reinforced. For video conference interviews, technical conditions and internet access were previously verified. In cases where offices had poor internet connection, or computers without cameras, a notebook with mobile broadband internet access was provided. Subsequently, a technical check of the internet connection, Zoom® platform functionality, and audio-visual quality was performed before formally starting the interview, which lasted between one hour and one hour and thirty minutes.

A semi-structured interview guideline was used with open-ended guiding questions that mentioned their daily life at work, their experiences, and their challenges. These questions included: What are the challenges you face in your work? Have you experienced or are you experiencing difficult, complex situations on an exceptional or permanent situation? Do you and your work team have contact with hazardous, polluting procedures or products? Do you have to perform excessive forces or movements that affect you physically? Please indicate the three risks you consider most serious for your teamwork. Is this severity due to physical, emotional, or psychological risk? Which one affects you most personally and why? 

Regarding the focal groups, six were held for the NTEs staff and five for clinical nurses in rooms designated by the participating hospitals. A total of 39 nurses and 44 NTEs participated in these activities. The focal groups were conducted by the occupational psychologists of the research staff, who alternated the roles of observer and moderator. The modality for the focal groups was hybrid due to the sanitary conditions of the pandemic and travel restrictions. This is why, according to the epidemiological situation of each day, focal groups were held with both psychologists present and focal groups with the moderator present and the observer remotely with a connection via the Zoom^®^ platform. Before starting the activity, an introduction to the study and its objectives was provided. As well as for the interviews, a semi-structured guideline with open questions was used addressing their working life, such as: What is your job, let's think about what are your daily tasks? With whom do you interact? In what setting does your work take place? What is your role within the teamwork? How is your relationship with your teamwork? How is your relationship with superiors? How is your relationship with the organization? Do you relate with patients? What is the challenge in this relationship? Do you work in shifts? What are the principal difficulties related to shifts?

Both focus groups and individual interviews were recorded in audio and video files, backed up and accessible only to the research team. Non-participant observation involved accompanying daily tasks and observing the working conditions in 30 clinical units (intensive care, adult and pediatric medical-surgical services, surgical wards, sterilization, dialysis, emergency, traumatology, endoscopy, post-anesthesia recovery, quality unit, unit for prevention of healthcare-associated infections, and psychiatric units). Field notes were taken using a field notebook, which included recording the narrative and meta-narrative of the observations. Subsequently, the notes were transcribed into a Word document for further review and analysis. The recordings of the interviews and focus groups were transcribed using the AmberScript® program and saved in a Word file. This initial transcription was then reviewed and corrected by the research team. To safeguard the confidentiality of participant identification data, an alphanumeric code was assigned to each interview. The data obtained from the discourses were initially analyzed using the predetermined categories and subcategories. The textual citations were grouped into first- and second-level categories and subcategories. Content from each code was analyzed, comparing it with the rest and identifying the elements in common, which permitted the creation of additional sub-categories to those previously defined.

During a second analysis stage, axial coding was carried out giving rise to the second-level categories. Finally, three instrument proposals were built: one for clinical nurses, one for nursing heads, and the other for NTEs, given the differences between their job profiles and their exposure to different hazards at work. 

Non-participant observation consisted in monitoring the daily tasks and observing the conditions in which the work was performed and this was conducted in 30 clinical units (intensive care, adult and pediatric medical-surgical services, surgical wards, sterilization, dialysis, emergency, traumatology, endoscopy, anesthetic recovery, quality unit, unit for the prevention of infections associated with health care, and psychiatry units). Field notes were taken in a field notebook that considered the record of the story and the meta-story of that observation. Thereafter, these were transcribed into a Word file for later review and analysis. The recordings of the interviews and focal groups were transcribed using the AmberScript^®^ program and stored in a Word file. Then, this first transcription was revised and corrected by the research staff. To safeguard the confidentiality of participants' identification data, an alphanumeric code was assigned to each interview. Thereafter, the data obtained from the discourses were subjected to analysis, using in the first instance the categories and subcategories defined *a priori*. The content of each code was analyzed and compared with the others, and common elements were identified, leading to the creation of additional subcategories beyond those defined beforehand.

In the second stage of analysis, axial coding was performed, resulting in second-level categories. Finally, three instrument proposals were developed: one for clinical nurses, one for nursing managers, and another for NTEs considering the differences in their job profiles and exposure to different work hazards.

### Stage 3. Quantitative study

The three instruments proposed were subjected to content validity by the judgment of 10 professional experts (two risk prevention professionals and eight nurses with a Master’s degree and experience of over ten years in intra-hospital work). The validity assessment was performed through criteria of relevance, conceptual clarity, and writing, using the methodology proposed by Hernández-Nieto.^23^ Next, a pilot study of a proposed instrument with 128 items was conducted on 15 nursing professionals from hospitals different from those studied. 

This pilot study revealed the impossibility of generating a common instrument for nursing managers, clinical nurses, administrative nurses, and NTEs (nursing technicians) due to the significant variability in positions, workplaces, types of responsibilities, and exposure to different risks. Although the requirement of the Occupational Safety Institute was to build an instrument, in practical terms, it was impossible to have a common instrument even differentiating among nurses managers, clinical nurses, and NTEs, given that the 128 items did not apply to all job types, therefore, three possible groups of questions were grouped: the first with common items for all the nurse and NTEs positions; the second with common items to all the nursing positions that care for patients; and the third with common items to nurses and NTEs who do not care directly for patients. However, the combination of the 128 original items can give rise to 17 instruments (two for nurses managers, eight for clinical and administrative nurses, and seven for NTEs) that consider the specific characteristics of each job position in the different clinical and administrative units and the hazards related with the direct patient’s care, such as biological, physical, chemical agents and others, like shift work. Differences in the administrative and leadership tasks and team leadership were also considered. 

This pilot study revealed the impossibility of achieving the initial purpose of constructing and validating a single instrument. Due to the magnitude and technical feasibility of validating 17 instruments, it was decided to subject the three instruments with common items to an initial study of validity and reliability, leaving for a later stage the validation of the 17 assessment tools foreseen. Then, the validity and reliability study was conducted, which was proposed to be carried out in the population of nursing workers from the four hospitals (n = 620). The research design corresponded to a quantitative study with descriptive cross-sectional scope carried out between December 2021 and April 2022. 

Data collection was carried out in each of the clinical and administrative services where nurses and NTEs worked. Two members of the research team delivered the instruments to the nursing staff in a sealed envelope that included the instrument and the informed consent to be self-answered. Then, the envelopes were collected on consecutive days until completing the data capture period. Since this was conducted during the summer period, it was necessary to wait for the workers' vacation periods. Therefore, both the delivery and retrieval of the instruments were done once a week in the four establishments.

For each group of interest (nursing managers, clinical nurses, administrative nurses, and NTEs), the corresponding instrument for the job position and unit in which each nurse and NTEs worked was used. This included a variable number of questions on a five-point Likert scale ranging from 1 = "strongly disagree" to 5 = "strongly agree". Once the instruments were collected, the responses were entered into an Excel spreadsheet, which was later exported to a data file in the statistical software Stata version 17, following the required data cleaning protocols before conducting any analysis. The following strategies were used: *Descriptive statistics*: while taking into account that Likert scale variables are qualitative and ordinal, the average and standard deviation were calculated for each item and dimension of the instrument. Additionally, the proportion of responses for each Likert scale in all items was calculated. *Internal consistency:* the reliability analysis of the items and categories was examined by calculating the α-Cronbach coefficient. *Construct validity:* to assess the coherence of the scale construction with the theoretical model, Kendall's correlation was used as a first step to applying exploratory factor analysis (EFA). The EFA was conducted using the principal axis extraction method with Varimax rotation, as this rotation method seeks to maximize the loadings at the factor level. In other words, each item or variable is expected to be represented in only one factor in order to minimize the number of variables in each factor. Additionally, the basic assumptions of sphericity were tested through Bartlett's test, complemented by the Kaiser-Meyer-Olkin (KMO) test. The KMO takes values between 0 and 1 and was performed to provide a measure of the adequacy of the factor analysis. If the values are small, it means that the items generally have very little in common to justify a factor analysis. Historically, the following labels are assigned to KMO values: 0.00 to 0.49 = unacceptable, 0.50 to 0.59 = miserable; 0.60 to 0.69 = mediocre, 0.70 to 0.79 = intermediate, 0.80 to 0.89 = meritorious, and 0.90 to 1.00 = excellent. Additionally, as a preliminary assessment of the goodness of fit of this factor analysis, standard goodness-of-fit indicators were calculated, excluding categories with only one item: Standardized Root Mean Square Residual (SRMR), Root Mean Square Error of Approximation (RMSEA), Tucker-Lewis Index (TLI). Values below 0.05 for SRMR and RMSEA indicate a good fit of the model, while values above 0.90 for the Tucker-Lewis Index and Comparative Fit Index indicate an adequate model fit. (24)

*Variable standardization:* The variability in the number of questions resulted in an imbalance in the structure of risk categories, which led to the need to standardize the scores to generate a single risk assessment indicator that would make the instruments comparable, regardless of the number of questions they included.

Taking into account the number of items per dimension in each instrument, the minimum and maximum risk scores were established. Then, the scores were centered in relation to the minimum score, as well as standardized according to the maximum score. The latter corresponds to the Overall Occupational Risk Index (OORI) and ranges from 0 to 100 points. The following cutoff scores were also established for three risk levels: low risk (0-39 points), medium risk (40-70 points), and high risk (71-100 points). This was done by applying the norm based on percentiles. (25) Finally, the scores obtained by the participants in each of the eleven risk categories were standardized, resulting in a Specific Occupational Risk Index (SORI).

Ethical considerations: Approval was obtained from the Scientific Ethics Committee of the Universidad Viña del Mar (CEC UVM 23/09/2020) and the Aconcagua Health Service (CEC 20/2020). Informed consent was obtained from all study participants

## Results

### Stage 1. Integrative literature review

As a result of the integrative review, 11 preliminary categories of risks were identified based on their nature: occupational stress and burnout, risks associated with the individual and their relationship with the organizational environment, mental workload, shift work, workplace violence, risks associated with the relationship styles of healthcare personnel or work teams, musculoskeletal risks, biological risks, physical risks, chemical risks, and double presence; along with 26 first-level risk subcategories. The predetermined categories and subcategories of occupational risks formed the basis for guiding the qualitative study in the second phase of this research.

### Stage 2. Qualitative study

The analysis of the discourse obtained from the interviews and focus groups led to the identification of the same eleven categories of risks resulting from the literature review. A total of 1355 textual citations were analyzed. The most frequently mentioned categories of occupational risks by the participants, in descending order, were: stress and emotional exhaustion, risks associated with the individual and their relationship with the organizational environment, mental workload, and relationship styles of healthcare personnel and work teams, representing 75.1% of the citations. It was observed that while the risk categories are common among most nurses and nursing technicians, they affect them differently, determining their way of experiencing and evaluating them. Furthermore, the nature of work in certain clinical units exposes professionals to quite specific chemical and physical risks. Therefore, the eleven categories of risks gave rise to 25 first-level subcategories and 98 second-level subcategories. Table 1 shows the classification of the categories and first-level subcategories.

**Table 1:** First-level categories and subcategories of occupational risks

First-level category and subcategory
*Stress and emotional exhaustion*
Work overload
Care and relationship with patients and relatives
Lack of self-care
Leadership concerns
Fear of contracting infectious diseases.
*Risks associated with the individual and their relationship with the organizational environment*
Perception of the lack of appreciation of nursing work
Inefficient management
Lack of training and formation
Insufficient staffing to perform the job
*Mental workload*
Capabilities and/or attitudes of people
Types of tasks and work methods
Equipment and infrastructure
Administrative aspects
Relationship among services and their link with patient health
*Shift work*
Perception of risk at work
*Violence in the workplace*
Violence associated with daily work
*Relationship styles of health personnel or teamwork*
Leadership
Communication within the staff
Work climate and dysfunctional dynamics
Teamwork
*Musculoskeletal risks*
Musculoskeletal risks associated with the nature of the work
*Biological risks*
Biological risks associated with the nature of the work
*Chemical risks*
Handling/exposure to chemical substances
*Physical risks*
Physical risks associated with the nature of the work
*Double presence*
Double presence associated with the nature of the work

**Figure 1:**
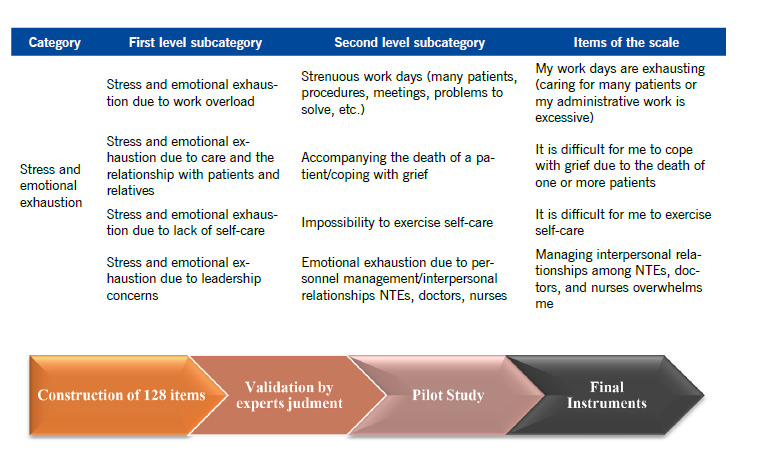
Scheme to elaborate the instrument’s questions. Example of the Stress and Emotional Exhaustion category and four items.

Based on the first- and second-level categories and subcategories, 128 items were constructed, which can give rise to 17 nursing team occupational risk scales (NTRS) with a different combination of questions, according to the job position and clinical or administrative unit with a varying number of questions depending on their position and work unit (nurses managers: between 64 and 81 questions, clinical and administrative nurses between 55 and 99 questions, and NTEs between 68 and 97 questions). These 17 differentiated scales should be validated in the future. (Figure 1) shows the construction process of the instrument’s items from the results of the qualitative study and stages prior to the instrument’s validity and reliability study.

### Stage 3. Quantitative study

The content validity analysis conducted by expert judgment resulted in a content validity coefficient of 0.97. Minor adjustments were made to some words based on their suggestions. Subsequently, the pilot study indicated the impossibility of validating a total of 17 instruments generated from the combination of 128 items. Therefore, a preliminary study of psychometric properties was conducted on three instruments with common items: one for all nursing positions and NTEs, another for nurses and NTEs who attend to patients, and finally, an instrument for nurses and NTEs who do not attend to patients. Out of the 620 instruments distributed, 512 were collected. However, nine were discarded due to a high number of omitted responses. Hence, a total of 503 valid instruments were included in the analysis, which was completed by nurses and NTEs from the four hospitals studied (430 who worked with patients and 73 who did not work with patients): 198 from San Juan de Dios Hospital in Los Andes, 210 from San Camilo Hospital, 60 from Philippe Pinel Hospital, and 35 from Llay Llay Hospital. Among the total sample, 31 corresponded to nursing management positions, 171 to clinical nurses (13 from administrative services, four from dialysis, 78 from adult and pediatric medical-surgical services, 12 from the operating room, five from traumatology, 38 from the critical care unit, 20 from the emergency department, and one from sterilization), and 301 to nursing technicians (seven from dialysis, 116 from adult and pediatric medical-surgical services, 42 from the operating room, 13 from traumatology, 34 from the critical care unit, 61 from the emergency department, and 28 from sterilization).

Given the variability in the number of risk categories and questions applicable to different nursing positions, three groups of items were created for an initial validation approach. The first group consisted of 52 items common to all positions, corresponding to eight risk categories. The categories "Shift work," "Chemical risks," and "Biological risks" were excluded from this analysis as they were specific to certain positions and clinical services. The second group incorporated nurses and NTEs who work with patients, sharing a total of 90 items corresponding to the eleven risk categories. Finally, the third group comprised nurses and NTEs who do not work with patients, sharing a total of 52 items corresponding to the same eight categories as the first group. For the first group including all positions and 52 common questions, Cronbach's alpha statistic was calculated for each item, resulting in an overall Cronbach's α of 0.9025. Table 2 shows that all items yielded values above 0.889, indicating excellent reliability of internal consistency for the 52-item instrument.^26^

**Table 2:** Cronbach’s alpha results for each item common to all nursing staff posts

Category/ items	Cronbach’s alpha
Stress and emotional exhaustion	
My workdays are exhausting	0.9001
I can rest outside working hours	0.9009
I have a work overload due to supervising new staff	0.9007
It is difficult for me to practice self-care	0.9006
There are formal instances of self-care	0.9015
There are informal instances of self-care	0.9013
I am aware that my self-care is important	0.9047
My responsibility and commitment go beyond the established	0.9043
I have been afraid of contracting infectious diseases	0.9023
Risks associated with individuals and their relationship with the organizational environment	
My work is valued by my supervisor	0.9032
My work is valued by the management	0.9006
The management pays little attention to how workers feel	0.9003
The hospital is efficient in problem-solving	0.8997
I participate in decision-making regarding my working conditions	0.9004
The management does not address occupational risk issues	0.8997
The management gives little importance to personnel problems	0.8997
Occupational risks-related problems are solved	0.9005
There is infrastructure without maintenance	0.9006
There is a shortage of healthcare staff	0.9015
Training is available for new personnel	0.9009
I have access to training for new challenges	0.9007
Mental workload	
I struggle to concentrate during the workday	0.9009
I feel I do not remember the things I have to do	0.9013
I have difficulty processing all the information	0.9008
It exhausts me when my colleagues resist changes	0.8999
It exhausts me when my superiors are resistant to changes	0.8992
The tasks I perform are highly demanding	0.9012
Continuous changes in procedures overwhelm me	0.8997
It overwhelms me to have to keep an eye on the work of new staff	0.8996
The distribution of resources among services is equitable	0.9007
We have the infrastructure for the nursing staff	0.9014
The management is only interested in our being productive	0.8993
My professional opinion is taken into account	0.9001
Established quality standards are met	0.9018
The conditions in which employees are hired are equitable	0.9000
I am exhausted by having to worry about mistakes that other members of my health staff could make	0.9002
Violence in the workplace	
I have suffered verbal aggression from a member of my work staff	0.9006
Healthcare staff’s relationship styles or team dynamics	
My supervisor exhibits good leadership	0.9008
Communication with my direct management is expedited	0.9014
Communication with my peers is effective	0.9016
The hospital´s management clearly communicates the organization´s general guidelines	0.9004
Part of the coexistence issues at work is due to teams comprising individuals of different ages or generations	0.9032
There are coexistence problems among employees in my department	0.9005
In this hospital, marked differences exist among the levels of the health staff	0.9011
Musculoskeletal risks	
There are conditions in my department that increase the risk of falls among personnel	0.9006
Physical risks	
I am exposed to extreme temperatures	0.9007
I am exposed to risks due to poor lighting	0.9004
I am exposed to risks due to infrastructure in bad conditions	0.8989
I am exposed to risks due to the lack of safety signage	0.8992
I am exposed to risks due to a lack of emergency protocols	0.8998
Double presence	
While I am working, I am worried about what is going on in my home	0.9012
In general, I can balance my work and personal life	0.9021
Overall Cronbach’s alpha	0.9025

The KMO test resulted in 0.8704, and Bartlett's test yielded a p-value less than 0.05, indicating the presence of correlation structure, which made it feasible to study the exploratory factor structure of the instrument, leading to conducting EFA. The Varimax rotation distinguished eight factors that grouped the items of the eight studied categories. The exploratory fit indices of the model were not satisfactory (TLI=0.535, RMSAE=0.096, RSAE=0.079). For the second group consisting of nurses and NTEs who attend to patients, the overall Cronbach's alpha was 0.932. All items resulted in values higher than 0.93, indicating excellent internal consistency reliability of the 90-item instrument.^26^ The KMO was 0.8417, and Bartlett's test was significant, demonstrating the feasibility of conducting EFA. It revealed a total of 11 factors that, like the previous case, grouped the items of the 11 occupational risk categories. Convergence was not achieved to evaluate the satisfaction of the goodness-of-fit indicators. Finally, for the third group composed of nurses and TENs who do not attend to patients, the overall Cronbach's alpha was 0.88. All items resulted in values higher than 0.87, indicating good internal consistency reliability of the 52-item instrument. ^26^ The KMO was 0.7469, and Bartlett's test was significant, allowing for the EFA, which identified nine factors that more clearly grouped the items of the eight common categories in this third grouping. The exploratory fit indices of the model were not satisfactory (TLI=0.167, RMSAE=0.163, RSAE=0.175).

## Discussion

Although the construction of a common occupational risk assessment instrument for nurses and NTEs was initially considered, the development of the qualitative phase and pilot study detected differences in the exposure to these risks, depending on the professional profile and workplace. This allows us to visualize for future research, specific instruments according to these criteria.

Since this project was carried out following guidelines provided by the state funding agency for the study, which stated that the instrument items should emerge from the initial qualitative study, it was not feasible to control the number of items, despite the suggestion of having a balanced number of items per dimension.^27^ However, the imbalance of these items highlights the importance of each category and reflects the working conditions faced by nursing staff. Out of a total of 128 items, not even 38 of these items could be incorporated into the preliminary validation process, as they were applicable to a very small number of professionals exposed to chemical and physical risks. This prevented us from having a sufficient quantity of the latter to carry out this initial validation process.

Regarding the question set of the NTRS, the dimension of stress and emotional exhaustion includes aspects that are experienced as risk factors. Within these factors, work overload, exhausting work schedules, and aspects associated with patient and family relationships are among the items investigated in this dimension, which is consistent with other authors.^1^ Although this research was conducted during the pandemic, the results indicated that COVID exacerbated the impact of risk factors rather than adding new ones, except for the fear of contagion. This has been described as a source of stress and anxiety among nursing professionals during a pandemic.^19^

Also, regarding the risks associated with the relationship between the individual and the organization, the items in this dimension reflect the perception of nursing work valuation, the impact of organizational decisions, and the lack of staff, among others, similar to what is described in the literature.^2^^,^^17^

In the mental workload category, the items reflect cognitive efforts to simultaneously handle a considerable amount of information and a multitude of tasks, many of which have high demands and must be completed within limited time periods. ^4^ The shift work dimension includes questions associated with the impact of shifts on health, personal life, and patient safety. These are consistent with studies that have detected issues such as eating and metabolic disorders, chronodisruption, anxiety, and stress, among others.^4^^,^^9^ Regarding risks associated with relationship styles, items on leadership, teamwork, and communication were incorporated, which are inherent to the nature of nursing work and have different effects depending on the role.^2^^,^^17^

Additionally, the instrument captured relevant aspects related to exposure to musculoskeletal risks, which is a complex issue as working conditions and risky activities can extend throughout a significant part of a professional's life, causing acute and chronic pathologies.^3^^,^^7^^,^^8^^,^^15^^,^^20^ The dimension of workplace violence included aggression from both healthcare personnel and patients and their families, as well as items related to the perception of institutional support in the face of aggression. This is a growing problem that affects the well-being of healthcare workers and poses challenges for professionals and institutions to manage.^20^ The items in the categories of physical, chemical, and biological risks refer to multiple agents capable of causing occupational diseases, with rigorous regulations that must be complied with to protect personnel, without finding agents different from those described in previous studies.^11^^-^^14^

Finally, the instrument includes items that account for dual presence, which is consistent with what has been reported in scientific literature.^2^^,^^17^ As recommended by international organizations, occupational health, and safety should be seen as an organizational objective and integrated into a continuous and systematic risk assessment system.^22^ In this sense, constructing an NTRS was a complex task given the multiplicity of risks encountered in the workplace and the various factors influencing them. The NTRS provides cohesive data regarding working conditions based on job profiles, which is a differentiating element compared to various instruments that do not discriminate. Moreover, the Overall Occupational Risk Index (OORI) and Specific Occupational Risk Index (SORI) provide understandable communication of a result that represents the level of risk, impacting both the prioritization and type of intervention measures to improve outcomes and the optimization of resource utilization. While the results generated by the instrument provide a general understanding of occupational risk issues, further exploration can be achieved by applying other validated instruments to evaluate each risk. This led to the construction of a set of 128 items that allowed, in this initial approach, the discernment of three instruments to assess occupational risks for the nursing team. The validation process should be continued to improve the construct validity in future research. However, an overall risk indicator and eleven specific risk indicators, according to risk categories have already been proposed. This constitutes a first step towards advancing the evaluation of risks for the nursing team and generating interventions to improve their well-being and work-related health.
